# Clustering Functional Magnetic Resonance Imaging Time Series in Glioblastoma Characterization: A Review of the Evolution, Applications, and Potentials

**DOI:** 10.3390/brainsci14030296

**Published:** 2024-03-20

**Authors:** Matteo De Simone, Giorgio Iaconetta, Giuseppina Palermo, Alessandro Fiorindi, Karl Schaller, Lucio De Maria

**Affiliations:** 1 Department of Medicine, Surgery and Dentistry “Scuola Medica Salernitana”, University of Salerno, Via S. Allende, 84081 Baronissi, Italy; m.desimone82@studenti.unisa.it (M.D.S.); g.palermo10@studenti.unisa.it (G.P.); 2 Neurosurgery Unit, University Hospital “San Giovanni di Dio e Ruggi, D’Aragona”, 84131 Salerno, Italy; 3 Division of Neurosurgery, Department of Medical and Surgical Specialties, Radiological Sciences and Public Health, University of Brescia, Piazza Spedali Civili 1, 25123 Brescia, Italy; alessandro.fiorindi@asst-spedalicivili.it (A.F.); l.demaria@unibs.it (L.D.M.); 4 Division of Neurosurgery, Department of Clinical Neurosciences, Geneva University Hospitals, Rue Gabrielle-Perret-Gentil 4, 1205 Geneva, Switzerland; karl.schaller@hcuge.ch

**Keywords:** glioblastoma, functional MRI, GBM imaging, brain activity, BOLD signal, GBM prognosis, personalized treatment

## Abstract

In this paper, we discuss how the clustering analysis technique can be applied to analyze functional magnetic resonance imaging (fMRI) time-series data in the context of glioblastoma (GBM), a highly heterogeneous brain tumor. The precise characterization of GBM is challenging and requires advanced analytical approaches. We have synthesized the existing literature to provide an overview of how clustering algorithms can help identify unique patterns within the dynamics of GBM. Our review shows that the clustering of fMRI time series has great potential for improving the differentiation between various subtypes of GBM, which is pivotal for developing personalized therapeutic strategies. Moreover, this method proves to be effective in capturing temporal changes occurring in GBM, enhancing the monitoring of disease progression and response to treatment. By thoroughly examining and consolidating the current research, this paper contributes to the understanding of how clustering techniques applied to fMRI data can refine the characterization of GBM. This article emphasizes the importance of incorporating cutting-edge data analysis techniques into neuroimaging and neuro-oncology research. By providing a detailed perspective, this approach may guide future investigations and boost the development of tailored therapeutic strategies for GBM.

## 1. Introduction

Gliomas are primary tumors of the central nervous system (CNS), which, on a global scale, maintain an unfavorable prognosis despite advancements in diagnostic and therapeutic methods over recent decades [1].

This group of tumors is highly heterogeneous, exhibiting distinct biological properties, prognoses, and treatment strategies. The classification and grading of gliomas have undergone significant evolution since their initial categorization in 1926 by Bailey and Cushing [2].

The modern categorization of gliomas based on the WHO Classification of CNS Tumors was most recently revised in 2021. Since the 2016 edition of the WHO classification, gliomas have been classified based not only on histopathologic features but also on molecular parameters [3]. In the 2021 edition, the classification of diffusely infiltrating gliomas in adults involves considerations of histology, isocitrate dehydrogenase (IDH) mutation status, and other significant molecular changes, leading to the identification of three primary types.

The focus of this study is on the third type, namely WHO grade 4, IDH-wildtype glioblastoma (GBM). GBM represents the most prevalent malignant primary brain tumor in adults. Tumors of this category are typically diffusely infiltrating, characterized by a high cellular density and pleomorphism, exhibiting mitotic activity, and featuring either microvascular proliferation, necrosis, or a combination of both [4]. Some other GBMs lack high-grade histologic features and otherwise contain a specific molecular alteration such as epidermal growth factor receptor (EGFR) amplification, telomerase reverse transcriptase (TERT) mutation, or chromosome 7 gain/chromosome 10 loss [5]. Therefore, the WHO defines GBM as a grade IV cancer that is typically malignant, mitotically active, and predisposed to necrosis.

To understand the social burden of the disease, the epidemiology of GBM must be primarily considered. GBM accounts for 14.5% of all CNS tumors and 48.6% of malignant CNS tumors. It has an annual incidence of about 1/33,330. The prevalence is estimated to be about 1/10,000. They can occur at all ages, but in 70% of cases, patients are diagnosed at an age between 45 and 70 years [6].

The treatment of GBM should be based primarily on aggressive resection; partial exeresis is ineffective and often a source of neurological deficits anyway; surgery is believed to favorably modify the prognosis if at least 80% of the volume of the tumor is removed [7]. Unfortunately, these conditions are not always achievable, and eloquent areas need to be well defined by suitable imagining techniques before surgery [8]. In terms of resection, a recent study analyzed the prognostic value of a glioblastoma surgical grading system based on residual contrast-enhancing (CE) tumors. Karschnia and colleagues collected data from 1008 patients with newly diagnosed IDHwt glioblastoma treated with radiochemotherapy and found that patients with a maximal CE resection had better outcomes than those with a submaximal resection or biopsy. In addition, an extensive resection of a non-CE tumor was associated with improved survival, defining a new category called “supramaximal CE resection”. The study suggests that these classification categories could be valuable for stratification in clinical trials and that the removal of a non-CE tumor beyond the boundaries of CE could offer additional survival benefits [9].

To date, despite diagnostic and treatment efforts, GBM remains an incurable disease with a median survival of 15 months [10]. The rise in new functional neurosurgical techniques such as LITT and FUS in certain settings represents an example of the operative directions aimed at improving the prognoses of these patients [11]. GBM exhibits aggressive behavior largely due to its inherent intratumoral heterogeneity, which includes diverse cell populations and components of the microenvironment. This heterogeneity influences key cancer-related functions. Although “tumor heterogeneity” encompasses both intertumor and intratumor differences, GBM in particular shows significant intratumor diversity, which impacts the prognosis and response to treatment [12]. Our review delves into this intratumoral heterogeneity, exploring its implications from histomorphology to imaging alterations. We argue for the need to embrace technological advances to better understand and address this heterogeneity in the management of GBM.

In fact, the objective of this study is to explore the potential and applications of clustering techniques applied to functional magnetic resonance imaging (fMRI) time-series data for the enhanced characterization of GBM. Recent research on neuron–cancer interaction has revolutionized our understanding of glioma and metastasis, revealing how tumors infiltrate intricate neuronal networks. Therefore, it is imperative to study tumor-associated neuronal dysfunction, the impact of cancer treatments on the central nervous system (CNS), and the interactions between the peripheral nervous system and various types of cancer. In particular, the role of exosomes in facilitating “wireless communications” between the nervous system and cancer cannot be overlooked [13]. We suggest that all these aspects can be effectively assessed with fMRI clustering by establishing an important bridge between the molecular knowledge of cancer and its clinical impact. The study aims to investigate how novel methods, such as clustering, can provide valuable insights into the diverse patterns within fMRI data associated with GBM to improve the understanding and characterization of this aggressive brain tumor.

## 2. Imaging of GBM

Because the presenting signs and symptoms of HGGs are similar to those produced by other aggressive primary and metastatic brain tumors, the clinical data should be supplemented with other information [14]. As a required next step, the purpose of neuroradiological diagnostics is to establish whether, in each clinical context, there is a brain tumor or another space-occupying disease, its site, and if the tumor directly infiltrates the parenchyma (intra-axial) or if it is external to this (extra-axial).

### 2.1. Traditional Imaging Tecniques

The first exam of choice remains computed tomography (CT). Tumor pathology is recognized by CT in over 95% of brain cancers. Exceptions are small tumors, such as micro-neuromas, and rare tumors that, while involving the parenchyma, remain isodense compared to this [15]. When a CT scan is performed as a first imaging evaluation, the main radiological findings of a GBM may be a large heterogeneous lesion with a mass effect on other CNS structures such as the ventricles or the brainstem and a contralateral midline shift [15].

An MRI scan will still have to be performed as a complementary exam to provide, in positive cases, a better radio-anatomic demonstration of the tumor already seen in CT [16]. With CT and MRI, in addition to the tumor lesion, changes in the parenchyma secondary to the tumor, such as peritumoral edema, the deformation of brain structures, and secondary hydrocephalus, may be recognized. In both TC and RM, it is necessary to complete the study with contrast for enhanced characterization. It is already well known that contrastographic enrichment is not specific to tumors because it is linked to an alteration of the blood–brain barrier (BBB) that can occur in different pathological situations. As for cancers, we need to remember how enhancement is quite characteristic in certain histological types, but this is not an absolute rule, and therefore extreme caution must be used for diagnosis. Classic examples of contrastographic enhancement are those of GBM and anaplastic astrocytoma [17]. Although contrast enrichment is not always an indicator of malignancy, as it is due to the vascular richness of the tumor, it can sometimes be used as a surrogate marker of disease burden [18] or even to make prognostic evaluations [19]. 

Moreover, enrichment is not exclusive to glioma but can also occur in meningioma, a generally benign extracerebral tumor. Therefore, in this setting, differentiating meningiomas with atypical conventional MRI findings from malignant intra-axial tumors can be difficult. Calculating regional cerebral blood flow (rCBV) ratios and constructing signal intensity–time curves may contribute to the differentiation of meningiomas from intra-axial tumors [20]. Other diseases may exhibit similar enrichment patterns to cancers; such examples are brain abscess or metastasis [21,22]. 

By considering visible density changes in CT and signal changes in MRI and correlating these findings with clinical data, high levels of accuracy can be achieved in the diagnosis already in vivo, but absolute certainty can only be obtained with an adequate histological examination [23].

Recently, numerous artificial intelligence (AI) models have been developed to extract additional information from standard imaging prior to and following surgery. This supplementary information can be utilized for diagnostic, treatment, and prognostic reasons [24]. 

### 2.2. MRI Modalities for GBM Characterization

Nowadays, the gold standard for the radiographic characterization of GBM remains MRI. Standard MRI protocols for the study of GBM must include native T1-weighted (T1w), contrast-enhanced (T1-CE), T2-weighted (T2w), and T2-fluid-attenuated inversion recovery (T2-FLAIR) sequences.

Over the last decade, advanced MRI modalities have been increasingly utilized to further characterize GBMs more comprehensively and to better choose management options. These include multiparametric MRI sequences, such as higher-order diffusion techniques such as diffusion tensor imaging (DTI) and MR spectroscopy (MRS). fMRI and tractography are increasingly being used to identify eloquent cortices and important tracts to minimize postsurgical neurological deficits [23].

According to the excitation sequence used for MRI, typical findings of GBM could be summarized as follows (Table 1). The axial gradient echo (GRE) image depicts multiple foci of hypointense signal “susceptibility artifacts” compatible with intratumoral blood products [25]. The axial FLAIR-weighted image demonstrates heterogeneous mass with a surrounding infiltrating signal abnormality, a “FLAIR envelope”. A “FLAIR envelope” is typically a manifestation of a combination of tumor infiltration and edema [26]. In the T1-CE axial image, there is heterogeneous irregular peripheral enhancement with a central nonenhancing area, consistent with necrosis. The introduction of higher-order diffusion techniques marks a significant advancement in guiding precise surgical strategies. One such technique, diffusion tensor imaging (DTI), utilizes the anisotropic nature of water molecule diffusion, particularly along the preferred direction, influenced by factors like fiber density, diameter, myelin integrity, and extracellular space characteristics [27]. Anisotropy, quantified through a tensor, is reduced in conditions like axonal damage or vasogenic edema. DTI employs specific parameters to calculate spatial variations, generating images that can be overlaid on anatomical ones. This method enables the reconstruction of white matter fiber courses in three dimensions, providing a 3D representation of tracts superimposed on morphological images [28]. Magnetic Resonance Spectroscopy (MRS) also differentiates hydrogen nuclei in brain tissue based on chemical bonding. MRS obtains spectra identifying macromolecules, and their concentrations aid in distinguishing cell populations and disease features. Peaks in the spectra correspond to unique resonance frequencies, while integral values reflect molecule concentrations in the clinical region. Specifically, the integral of each peak reflects the concentration of the molecule in the region of clinical interest [29]. The proton spectrum of brain parenchyma features three crucial peaks: choline (linked to cell membrane synthesis), creatine (reflecting cellular energy metabolism), and N-acetylaspartate (indicative of neuron and axon integrity). In brain tumor studies, the choline peak, typically the first in the spectrum, is significant. This peak, encompassing choline from various sources, serves as an indicator of membrane turnover and increases in neoplastic processes or inflammation. The clinical context and additional imaging features are essential for a precise differential diagnosis [30]. All possible imaging techniques, and in particular specific MRI sequences, are being integrated into increasingly standardized protocols in order to make the results obtained in clinical trials reproducible and comparable. The International Standardized Brain Tumor Imaging Protocol (BTIP) minimum image acquisition requirements for 1.5 T and 3 T MR systems [31] and the aforementioned recent update of the Response Assessment in Neuro-Oncology (RANO) criteria [9] are undoubtedly hallmarks that move in this direction. Both, in fact, suggest significant improvements for response assessment in glioblastoma cases. These include the incorporation of a volumetric assessment of response, the use of contrast-enhanced T1 subtraction maps to improve lesion visibility, the elimination of qualitative tumor assessment requirements that do not increase, the shifting of the baseline for response assessment in newly diagnosed glioblastoma to the postirradiation time point, and the introduction of “treatment-agnostic” response assessment rubrics. These changes aim to better identify pseudoprogression, pseudoraponse, and a confirmed enduring response in both newly diagnosed and recurrent glioblastoma studies, and we also suggest that such a stratification may also be useful in clustering.

## 3. History of a Revolution

During the late 19th century, Angelo Mosso, an Italian physiologist, invented the “human circulation balance”, which could noninvasively measure the redistribution of blood during emotional and intellectual activity. However, the details and precise workings of this balance and the experiments Mosso performed with it remained largely unknown [39].

The next step in resolving how to measure blood flow to the brain was Linus Pauling’s and Charles Coryell’s discovery in 1936 that oxygen-rich blood with hemoglobin (Hb) was weakly repelled by magnetic fields, while oxygen-depleted blood with deoxyhemoglobin (dHb) was attracted to a magnetic field.

The Blood Oxygenation Level-Dependent (BOLD) signal is an MRI contrast signal of dHb, discovered in 1990 by Ogawa. In a seminal 1990 study based on earlier work by Thulborn et al., Ogawa and colleagues scanned rodents in strong-magnetic-field (7.0 T) MRI. To manipulate blood oxygen levels, they changed the proportion of oxygen the animals breathed. As this proportion fell, a map of blood flow in the brain was seen in the MRI scan. They verified this by placing oxygenated or deoxygenated blood in test tubes and creating separate images. They also showed that gradient echo images, which depend on a form of loss of magnetization called T2* decay, produced the best images. They changed the composition of the air breathed by rats and scanned them while monitoring their brain activity with EEG, showing that these blood flow changes were related to functional brain activity [40]. Starting from this cornerstone evidence, in the next few years, three studies were the first to explore using the BOLD contrast signal in humans. Kenneth Kwong and colleagues, using both gradient echo and inversion recovery echo-planar imaging (EPI) sequences at a magnetic field strength of 1.5 T, published studies showing the clear activation of the human visual cortex [41]. 

### BOLD fMRI

In clinical practice, fMRI using the BOLD technique exploits the speed and sensitivity to paramagnetic effects of Eco-Planar Imaging sequences to evaluate the changes induced on the magnetic field through the activation of “eloquent” areas of the brain. When an area of the brain is activated by a stimulus (task), it increases brain loco-regional blood flow and, with it, the wash-out of dHb (a paramagnetic substance) in favor of an increase in Hb (diamagnetic substance); as a result, the signal intensity increases, allowing the functional analysis software to recognize the activated areas versus the surrounding silent areas.

fMRI has become widely used in neurophysiological research and has enabled extraordinary progress in our knowledge of the brain’s functioning. In a clinical setting, functional studies are used to highlight the sensorimotor, speech, visual, and memory areas, generally in patients who must undergo resections of tumors adjacent to eloquent areas. fMRI has been particularly useful in preoperative neurosurgical planning in all cases where a tumor’s resection may disrupt eloquent areas or other anatomic–functional areas [42]. 

Many patients who were once considered unresectable due to the uncertain risk of neurologic compromise are now candidates for more aggressive resection after functional mapping [43].

To improve surgical planning, diffusion techniques, including DTI, generate rich white matter tractography images [44] and can help distinguish between postoperative vascular damage and residual MRI T1-enhancing tumors.

A frequency domain analysis of fMRI activity provides prognostic information in patients with GBM and offers a means to noninvasively study the effects of GBM on the whole brain. Indeed, one study analyzed the alterations in metabolism and brain-wide effects of GBMs using fMRI. Park et al. compared 189 patients with newly diagnosed GBM with a matched healthy reference group. The results showed significantly flatter spectra and a reduced gray matter fractional amplitude of low-frequency fluctuations in the patients compared with the reference group. The spectral changes were associated with a global dysregulation of excitatory and inhibitory balance and metabolic demand in a tumor-affected brain. In addition, clinical comorbidities, particularly seizures, and MGMT promoter methylation were associated with flatter spectra. The degree of variation in the spectra was predictive of overall survival [45]. 

A valuable application of resting-state (rs) fMRI in comprehensive presurgical evaluations and its potential to increase the accuracy of glioma delineation and improve surgical outcomes are offered by studies exploring the use of presurgical fMRI to detect gliomas, introducing a novel method based on independent component analysis (ICA) of rs-fMRI. The research includes data from 32 glioma patients at three centers and additional proof-of-concept data from 28 patients with nonbrain musculoskeletal tumors. Using a variable number of total components (TNCs) in individual ICAs, a template-matching algorithm automatically identifies tumor-related components. The success rates for glioma tissue detection are impressive: 100%, 100%, and 93.75% for the three centers and 85.19% for musculoskeletal tumor detection. The study suggests that these high success rates can be attributed to the ability of BOLD rs-fMRI to characterize abnormal vascularization, vasomotion, and perfusion caused by tumors [46]. 

## 4. Clustering

Grouping objects is required for various purposes in different areas of engineering, science, and technology; the humanities; medical science; and our daily lives. It is a technique used in data analysis and machine learning (ML) to group similar data points based on certain features or characteristics. The task of grouping a set of objects in such a way that objects in the same group are more similar to each other than to those in other groups is called clustering [47]. Cluster analysis was developed in anthropology by Driver and Kroeber in 1932 [48] and introduced into psychology by Joseph Zubin in 1938 [49] and Robert Tryon in 1939 [50]. It was made famous by Cattell, who used it to classify traits in personality psychology from 1943 [51]. 

The main purpose behind the study of classification is to develop a tool or an algorithm that can be used to predict the class of an unknown object that is not labeled. This tool or algorithm is called a classifier. The objects in the classification process are more commonly represented by patterns. A pattern consists of several features.

Clustering itself is not one specific algorithm, but a general task to be solved. It can be achieved by various algorithms that differ significantly in their understanding of what constitutes a cluster and how to efficiently find them. The appropriate clustering algorithm and parameter settings depend on the individual dataset and the intended use of the results; it is an iterative process of knowledge discovery or interactive multi-objective optimization. The classification accuracy of a classifier is judged by the number of test patterns it has classified correctly [52]. Fraley and Raftery suggested dividing clustering approaches into two different groups: hierarchical and partitioning techniques [53].

Hierarchical clustering is a clustering approach that aims to build a hierarchy of clusters. Strategies for hierarchical clustering are of two types: (1) agglomerative: this is a “bottom–up” approach in which one starts by placing each element in a different cluster and then gradually merges the clusters two by two; (2) divisive: this is a “top–down” approach in which all elements are initially in a single cluster that is gradually subdivided recursively into sub-clusters [54,55]. 

Partitional clustering is the opposite of hierarchical clustering; in this case, data are allocated in k-clusters without any hierarchical structure by optimizing some criterion function [56].

The most commonly used criterion is the Euclidean distance, which finds the minimum distance between points in each of the available clusters and assigns the points to clusters. According to the “No Free Lunch” concept given by Wolpert and Macready [57], no algorithm can be uniformly good under all circumstances. 

Table 2 summarizes the main characteristics of these two types of clustering.

In the context of medicine and healthcare, clustering has various applications, contributing to the understanding, diagnosis, and treatment of diseases. One of these is image segmentation in imaging, a fundamental technique that uses the concept of clustering to improve the interpretation and analysis of complex visual data. Clustering involves grouping pixels with similar characteristics, thus enabling the segmentation of an image into distinct regions. This approach proves particularly valuable in the context of brain lesions, where the identification and precise delineation of specific structures or abnormalities within images are critical for accurate diagnosis and effective treatment planning [61].

Clustering algorithms play a crucial role in this setting. By dividing an image into regions with similar pixel intensities or other relevant features, clustering facilitates the isolation of anatomical structures or pathological findings [62]. This process is critical in helping healthcare professionals understand normal and pathological morphology, detect abnormalities, and formulate personalized treatment strategies [63].

The advantages of clustering in medical image segmentation are many. First, it improves the accuracy of visual interpretation by isolating specific structures, reducing the risk of oversight. Second, it facilitates a more precise localization of abnormalities, aiding early diagnosis and characterization [64].

Despite its advantages, the application of clustering in medical image segmentation poses challenges. Sensitivity to noise and outliers, determining the optimal number of clusters, and managing computational complexity are among the issues that researchers and practitioners continually face. Ongoing research explores the integration of clustering with deep learning approaches to overcome these challenges, promising more accurate and efficient segmentation in the future [65].

In conclusion, the use of clustering represents a powerful synergy between data analysis and healthcare. This methodology provides medical professionals with better tools for visual interpretation, diagnosis, and treatment planning, contributing to the advancement of patient care and medical research. As technology continues to evolve, the integration of clustering techniques is set to play an increasingly integral role in shaping the future of medical imaging.

The study of functional connectivity is also useful in other settings, such as postCOVID syndrome, to characterize the organic substrate of COVID-related consciousness disorder [66].

## 5. The Role of Clustering fRMI Time Series in GBM

The real challenge in studying GBMs remains the heterogeneity within the spectrum of these tumors, making it difficult to delineate profiles of tumors with repetitive and predictable characteristics for practical clinical use [67]. However, several parameters can be considered to subtype different GBM profiles, such as molecular mutations, the utilization of clinical data (such as treatment responses, patient survival, and age), the use of clustering techniques, and others. To ensure that these approaches are validated, it is then necessary to perform cross-validation to confirm the consistency of the identified subclasses, for example, by dividing the dataset into training and test sets and subsequently leveraging machine learning approaches [68].

### 5.1. Characteristics of Gliomas in fMRI

fMRI can provide valuable information on the functional characteristics of gliomas and on how their presence modifies peritumoral brain activity, and, again, it provides useful information in surgical planning, evaluating the response to chemotherapy (CMT) therapy, in the prognostic evaluation of the patient, and, finally, in the evaluation of the risk of recurrence. Common features of gliomas in functional MRI include the following:

- Changes in the BOLD signal, which may reflect changes in local neuronal activity and vascular reactivity in and around the tumor area;
- Functional activity in the resting state: gliomas can modify their activity in the resting state, leading to alterations in brain networks associated with motor, linguistic, and cognitive functions;
- Functional activity in response to tasks: gliomas can also influence activation patterns in response to cognitive and motor tasks, leading to changes in the location and magnitude of brain activation [37].

Kumar et al. highlight the importance of standardizing the acquisition parameters of these metabolic techniques to facilitate their use in clinical practice. Their study highlights that cellular and metabolic alterations in brain tumors begin long before the appearance of actual lesions or anatomical changes, and these emerging techniques enable the early detection of such alterations. Although fMRI can provide valuable information on the functional characteristics of gliomas, it is not generally used to predict tumor behavior, as these aspects do not directly reflect tumor growth. To obtain such information, fMRI can and should be combined with other imaging modalities, such as DTI and perfusion MRI [37].

However, a more recent study from 2023 by Ki Yun Park et al. is proactive in the use of total brain activity visualized through fMRI for the prognostic evaluation of patients. They highlight that a flatter spectrum is unambiguously attributable to the global dysregulation of excitatory and inhibitory balance and metabolic demand in the tumor-bearing brain. Furthermore, clinical comorbidities, particularly seizures and MGMT promoter methylation, were also associated with flatter spectra (*p* < 0.005). In particular, this study suggests that a frequency domain analysis of resting-state (rs) fMRI activity provides prognostic information for patients with GBM and offers a means to noninvasively study the effects of GBM on the entire brain [45].

### 5.2. Predicting Responses to CMT and RT through fMRI Clustering

fMRI therefore appears to also play an important role in predicting response to CMT, by allowing for an assessment of tumor perfusion, angiogenesis, and metabolic activity. Regions of the tumor that are more metabolically active or have higher blood flow, known as regions of interest (ROIs), may be more resistant to CMT and may require alternative treatment strategies. Other factors that may contribute to resistance to CMT include the presence of hypoxia or necrosis [69].

This has led to the search for various markers which could be predictive of patient outcomes or treatment response. Several clinical and therapeutic factors, as well as specific tumor characteristics and histopathological and genetic markers, have been studied as potential prognostic markers of survival [69].

In a study by Rockne et al., a patient-specific, biology-based mathematical model is applied for glioma growth and invasion that quantifies the response to radiation therapy in individual patients in vivo. The authors point out that the response to radiation in these patients, quantified by both clinical and model-generated measures, could have been predicted before treatment with high accuracy. Specifically, the net proliferation rate correlated with the radiation response parameter (r = 0.89, *p* = 0.0007), resulting in a predictive relationship that is tested with a “leave-one-out” cross-validation technique. The results of this study suggest that a mathematical model can create a virtual tumor in silico with the same growth kinetics as a particular patient and can not only predict the response to treatment in individual patients in vivo but also provide a basis for evaluating the response in each patient to any therapy [70].

A simple pattern of GBM growth, calculated at the time of the first postradiotherapy (RT) MRI scan, may therefore be prognostic for time to tumor recurrence and overall patient survival [71].

However, it is necessary to remember that it is not so much the size of the GBM that impacts prognosis but the tumor growth kinetics (TGK). If the tumor proliferates faster, it is likely to be larger at diagnosis, but at the same time, it will respond more to adjuvant CMT, leading to longer survival. A predictive model was developed to identify which cluster a patient would likely fall into based on postRT information, with an accuracy of 90.3%. Although the tool used is not fRMI, this study confirms that one parameter on which we can base a prediction of response to therapy is tumor growth kinetics [72].

However, previously, we discussed how an increase in the BOLD signal in fMRI is associated with a better prognosis. We also mentioned that the BOLD signal reflects changes in local neuronal activity and vascular reactivity [37] and that tumor growth kinetics correlate with an increased BOLD signal [72].

If growth kinetics correlate with the probability of response to therapy (either RT or CMT), we infer that fMRI can predict responses to therapy. The higher the signal in BOLD, the greater the hope that the patient will respond to treatment. Therefore, the use of fMRI is proposed for stratifying GBMs according to neuronal activity in different clusters to predict responses to treatment.

### 5.3. Clustering Genetic Mutations and Advanced Imaging in Glioblastoma Multiforme (GBM) for Personalized Treatment Strategies

Techniques such as functional diffusion mapping (fDM) have been used to track treatment responses in gliomas and have demonstrated the ability to predict responses to both CMT and targeted therapies [73].

Knowing today that genetic mutations of a tumor correlate with the aggressiveness of that tumor and the kinetics of proliferation, we can think of also including in the different clusters of GBM the underlying genetic mutation, which then not only can help us to predict the probability of responses to therapy but also what the best therapy is to achieve the most personalized therapy possible [74].

Bearing in mind, however, that GBM is characterized by profound intratumoral heterogeneity and thus correlates in a complex manner with tumor growth kinetics visualized in fRMI, this aspect is still a limitation today [75].

MRI can also be used to guide a biopsy or surgery, both preoperatively and postoperatively. By mapping functional areas of the brain, fMRI can help surgeons plan tumor resections while preserving critical brain functions such as language and motor skills. In addition, fMRI can be used to assess changes in brain function after surgery, providing information about a patient’s recovery and potential long-term prognosis [74].

Another use of MRI, both classic and functional, is the early evaluation of the response to treatment. MRI, with the aid of spectroscopy, can detect metabolic changes within tumor tissue, such as alterations in choline, N-acetylaspartate, and creatine levels, which may indicate an early response to treatment. Whole-brain spectroscopic MRI biomarkers can be used to identify infiltrating margins in GBM patients, potentially indicating an early response to treatment [37].

A study by Pope et al. suggests that ADC histogram analyses can potentially serve as a valuable tool for predicting tumor responses to anti-angiogenic therapy, particularly in newly diagnosed GBM patients. The use of an ADC histogram analysis may enable a more personalized approach to treatment, allowing doctors to identify patients who are most likely to benefit from bevacizumab therapy based on tumor characteristics [76].

### 5.4. fMRI and Clustering Algorithms for Improved Diagnosis and Treatment Stratification

Despite the uncertainty in the reliability of the role of fMRI in predicting relapse, several studies have described the reliability of fMRI in the diagnosis of relapse. Khan et al. report a pilot study that investigated the efficacy of restriction spectrum imaging (RSI) in distinguishing between tumor recurrence and treatment effects in patients with GBM undergoing radiation–temozolomide (RT/TMZ) therapy, achieving a sensitivity of 84% and a specificity of 86% in detecting tumor recurrence, with a positive predictive value of 95% (a high probability of correctly identifying true positives) and a negative predictive value of 60% (suggesting that although negativity of the RSI may not definitively exclude recurrence, it is nevertheless valuable in evaluating the absence of tumor cells). The potential benefits of RSI include increased sensitivity in detecting tumor recurrence and treatment effects, as well as an improved correlation with histopathological findings. The implications for the clinical management of GBM patients undergoing RT/TMZ therapy include the potential for RSI to serve as a valuable tool for differentiating between treatment effects and tumor recurrence. This could lead to more informed decision making and better clinical outcomes for patients [77]. The identification of these parameters listed so far is necessary but not sufficient for delineating GBM profiles. To achieve this, the use of appropriate clustering methods is necessary, such as k-means or other clustering algorithms. The most commonly used algorithms in medicine include k-means, hierarchical clustering, DBSCAN (Density-Based Spatial Clustering of Applications with Noise), Spectral Clustering, and Gaussian Mixture Models (GMMs) [52].

K-means clustering, in particular, can be leveraged to segment medical images of the brain obtained from fMRI to help identify different regions of a tumor or distinguish tumor lesions from healthy tissues; classify tumor subtypes by analyzing molecular data and radiomic features; assess treatment response; and ultimately identify biomarkers associated with specific tumor characteristics or treatment responses. These biomarkers could be used to personalize therapies based on a tumor’s characteristics [78,79,80].

## 6. Future Directions

This study aims to be an opportunity for reflection for scientists and a point of arrival for scientific research, as it is now clear that fMRI can provide additional and nontrivial data, compared to other methods for imaging, about tumor behavior [81]. In fact, by providing parameters regarding tumor perfusion, angiogenesis, and metabolic activity, which can indirectly be predictive indices of responses to CMT and RT, it turns out to be a tool that can be used for the global prognostic assessment of patients [82].

Research in the near future will probably be directed towards the integration of fMRI with other advanced imaging techniques, such as diffusion imaging and MR spectroscopy, which could improve the precision of the localization and characterization of lesions.

Another research direction could be the in-depth study of neuroplasticity in patients with GBM and therefore the brain’s ability to adapt to structural changes induced by a tumor and posttherapy outcomes, which could be studied to develop targeted rehabilitation interventions. Our study also hopes to be a solid starting point for future scientists who, starting from this evidence, can strengthen the statistical significance of the data provided by fMRI. We propose that in the future, by starting from the already known mutational profiles of various GBMs and studying the behavior of each of them through fMRI, we can identify imaging parameters that can, by themselves, help doctors outline personalized therapies for patients [83].

## 7. Issues and Limitations

fMRI is a powerful technique; however, it has some limitations and challenges. It has a limited temporal resolution, typically of the order of seconds. To capture a change in brain activity, the phenomenon must persist for at least a few seconds; otherwise, it is not captured at all, making it difficult to distinguish very fast neural events [84].

At the same time, it naturally has spatial limitations, as it cannot capture the activity of individual neurons but only that of contiguous groups of neurons [85].

An important limitation is inherent in the rationale on which fMRI is based: the signals detected are determined by changes in blood flow, oxygenation, and volume. Therefore, it is difficult to distinguish between specific neuronal activity and associated vascular changes [86].

There are also procedural limitations: fMRI is sensitive to subject movements, and even small movements can introduce artifacts into the data. Furthermore, access to fMRI facilities can be limited, especially in certain regions or institutions [87].

Other limitations pertain specifically to the use of parameters obtained from fMRI. Despite the awareness of the importance of fMRI and the valuable information it provides, it is currently not possible to identify the precise profiles of GBMs with repetitive and predictable behaviors through fMRI. There are still no specific biomarkers related to changes in BOLD signals that, on their own, can uniquely predict a tumor’s behavior. One idea could be to identify repetitive and predictable behaviors through fMRI and associate them with the specific mutational profiles of GBM; however, it is important to keep in mind that different mutations coexist simultaneously in a tumor mass [67].

This heterogeneity may represent a limit to the identification of a “typical tumor” and its use as a prediction model.

Additionally, the k-means algorithm itself has some limitations; for example, it assumes that clusters have a spherical shape and similar sizes; however, in fMRI data, clusters might have more complex shapes or vary in sizes, leading to suboptimal results. The choice of the number of clusters, which is often arbitrary, can also significantly influence k-means results. fMRI data can exhibit considerable variability among subjects. Improper handling by the k-means algorithm might group subjects into clusters reflecting individual differences rather than common patterns. fMRI data are often multivariate, involving multiple brain regions and voxels [88].

Interpreting the clusters obtained from k-means can be complex, especially in fMRI data, where the clusters may not directly correspond to known anatomical structures or brain functions. K-means assumes that data points within the same cluster are homogeneous. However, in fMRI data, brain regions can have complex subpopulations, and this assumption may not always hold. To address some of these limitations, various strategies can be employed, such as using more advanced clustering algorithms, more accurate data preprocessing, and cross-validating results. Additionally, the integration of statistical approaches and machine learning techniques could help overcome some of the challenges associated with fMRI data analysis [89,90].

## 8. Conclusions

The study of GBM is highly complicated due to its high heterogeneity and aggressiveness. This heterogeneity makes precise tumor characterization and the definition of predictable tumor behaviors challenging. Over the years, scientists have sought molecular, radiomic, clinical, and laboratory parameters to categorize potential tumor subtypes for better study.

In this study, we review the use of clustering algorithms in the context of GBM. The rationale for using such algorithms is to subtype groups of GBM, allowing for a better understanding of tumor complexity for personalized treatment, therapy response monitoring, disease progression monitoring, and recurrence monitoring.

The use of clustering algorithms, specifically k-means, in GBM research aims to identify, characterize, and distinguish various subpopulations of tumor cells to enhance the understanding, management, and treatment of this complex brain neoplasm. However, we emphasize the importance of integrating advanced analytical methods in neuroimaging, such as diffusion imaging and MR spectroscopy, to obtain a more informative perspective that could guide future investigations and lead to the development of targeted therapeutic strategies for GBM. At the same time, we highlight how clustering analyses of fMRI time series can offer promising results in improving discrimination between different GBM subtypes.

## Figures and Tables

**Table 1:** Characteristics of GBM in MRI.

MRI Sequence	Characteristics
T1-Weighted Imaging [32]	- Hypointense or isointense appearance - Enhancement after contrast administration (ring-enhancing)
T2-Weighted Imaging [33]	- Hyperintense signal intensity - Presence of peritumoral edema - Ill-defined, infiltrative borders
FLAIR [26]	- Hyperintense signal, highlighting peritumoral edema - Improved visualization of tumor boundaries
Diffusion-Weighted Imaging (DWI) [27]	- Restricted diffusion, high signal intensity within the tumor - Useful in distinguishing tumor from surrounding tissues
Perfusion Imaging [34]	- Increased perfusion within the tumor - Assessment of neovascularization
Magnetic Resonance Spectroscopy (MRS) [35]	- Elevated choline peak - Reduced N-acetyl aspartate (NAA) and creatine peaks - Presence of lactate and lipid peaks
Postcontrast Imaging [36]	- Intense and heterogeneous enhancement - Central necrosis with a ring-enhancing pattern
Functional MRI (fMRI) [37]	- May provide information on tumor location and its impact on functional areas of the brain
3D Imaging [38]	- Useful for precise visualization of tumor morphology - Aids in surgical planning and guidance

**Table 2:** Characteristics of hierarchical and partitioning clustering techniques.

Characteristic	Hierarchical Clustering	Partitioning Techniques
Nature of Clusters [58]	Creates a tree-like structure (dendrogram).	Divides data into nonoverlapping clusters.
Types [59]	Agglomerative and divisive.	K-means, Partitioning Around Medoids (PAM), Fuzzy C-Means (FCM), etc.
Interpretability [52]	Provides a visual dendrogram for interpreting relationships between clusters at different levels.	Generally simpler to interpret but lacks the hierarchical structure.
Computational Complexity [60]	Can be computationally expensive, especially for large datasets.	Generally more computationally efficient, especially for large datasets.

## Data Availability

Not applicable.
